# Adherence to a Mediterranean Diet for 6 Months Improves the Dietary Inflammatory Index in a Western Population: Results from the MedLey Study

**DOI:** 10.3390/nu15020366

**Published:** 2023-01-11

**Authors:** Jessie S. Clark, Kathryn A. Dyer, Courtney R. Davis, Nitin Shivappa, James R. Hébert, Richard Woodman, Jonathan M. Hodgson, Karen J. Murphy

**Affiliations:** 1 Clinical and Health Sciences, University of South Australia, Adelaide, SA 5001, Australia; 2 Alliance for Research in Exercise, Nutrition and Activity, School of Health Sciences, University of South Australia, Adelaide, SA 5001, Australia; 3 Cancer Prevention & Control Program, University of South Carolina, Columbia, SC 29208, USA; 4 Department of Epidemiology & Biostatistics, University of South Carolina, Columbia, SC 29208, USA; 5 Flinders Centre for Epidemiology and Biostatistics, Flinders University, Adelaide, SA 5001, Australia; 6 Nutrition & Health Innovation Research Institute, School of Medical and Health Sciences, Edith Cowan University, Perth, WA 6027, Australia

**Keywords:** Mediterranean diet, DII^®^, MedLey, Western, cardiovascular outcomes

## Abstract

Increasing evidence supports that a higher dietary inflammatory index (DII^®^) score is associated with inflammation and cardiovascular disease (CVD) risk, events, and mortality. This randomized trial sought to determine if a change to a Mediterranean diet resulted in a reduction in the DII score, and then it evaluated the relationship between the DII and cardiometabolic outcomes following the administration of a traditional Mediterranean diet in older Australian adults. A total of 152 Australian adults (mean age 71 ± 5 years) was randomly allocated either a MedDiet (*n* = 80) or to continue their habitual diet (HabDiet) (*n* = 72) for 6 months. Diet and cardiovascular outcomes were measured at baseline and 3 and 6 months of the intervention. DII and energy-adjusted DII (E-DII^TM^) scores were calculated from 3-day weighed food records. There was a significant reduction in the DII score at 2 and 4 months for the MedDiet group (−1.40 ± 0.20 *p* < 0.001 and −1.47 ± 0.20 *p* < 0.001, respectively), which was significantly different from the HabDiet group over time (*p* < 0.001). The HabDiet DII score did not change significantly at the 2 and 4 months timepoints (0.47 ± 0.21 *p* = 0.35 and 0.54 ± 0.21 *p* = 0.21, respectively). The improvement in the DII in the MedDiet group was not related to any cardiometabolic outcome. Baseline cross-sectional analyses identified a positive association between the E-DII score and average BMI, body weight, WHR, abdominal adiposity, and SBP, and a negative association with HDL-C. We demonstrate that a MedDiet intervention significantly reduced DII scores compared with a habitual Australian diet in older Australians. This could be beneficial for healthy ageing and the avoidance of chronic disease in Western populations.

## 1. Introduction

The inflammatory response involves the production of a range of pro-inflammatory biomarkers including cytokines, interleukins (IL), adhesion molecules, tumour necrosis factor, and high sensitivity C-reactive protein (hsCRP) as part of the body’s acute natural defence to a potentially harmful stimulus. However, the continuous and sustained production of such pro-inflammatory compounds results in a state of chronic inflammation, which can be damaging to the body. Chronic inflammation is suggested to be an underlying pathophysiological risk factor for a range of chronic diseases, including cancers [1,2], type 2 diabetes, obesity, metabolic syndrome (MetS) [3], atherosclerosis, and cardiovascular disease (CVD) [4,5]. There is sufficient evidence supporting the ability of diet to modify chronic inflammation. Western-style dietary patterns, characterised by high intakes of added sugars, salt, processed meats, and unhealthy fats, have been shown to increase pro-inflammatory biomarkers such as hsCRP and IL-6 [6,7]. In contrast, plant-based diets rich in vegetables, fruit, wholegrains, nuts, and olive oil such as the Mediterranean diet (MedDiet) have been shown to reduce inflammation [8,9]. Further, the MedDiet may in part play a preventive role in cardiometabolic diseases and CVD-related mortality via its anti-inflammatory effect related to high concentrations of bioactive components, including micronutrients such as polyphenols in olive oil. 

In 2009, an evidenced-based tool was created, which was later updated and validated with the ability to categorize an individual’s diet as either pro-inflammatory or anti-inflammatory [10,11]. A pro-inflammatory diet as evidenced by higher dietary inflammatory index (DII^®^) scores is associated with CVD-related mortality [12], whilst an anti-inflammatory dietary pattern with a low DII score reduces CVD-related mortality, obesity, and risk of MetS [13,14,15,16]. In view of revising dietary guidelines and recommendations of dietary patterns for the prevention of chronic disease, an index that categorises the inflammatory potential of a diet could be an extremely useful tool in research and clinical practice to not only evaluate dietary patterns but to individualise treatment strategies. The primary aim of this study was to determine the effect of a 6-month MedDiet intervention on DII and E-DII scores, with secondary exploration of the relationship between the DII^®^ and cardiovascular risk factors, following the administration of a traditional MedDiet in the Mediterranean diet for cognition and cardiovascular health in the elderly (MedLey) trial in older Australians. 

## 2. Methods

The MedLey study was a dietitian-led, randomized, controlled, parallel dietary intervention trial comparing the effect of a traditional MedDiet with a habitual diet on cognitive and cardiometabolic health outcomes in a healthy elderly population. The protocol has been described elsewhere [17,18]. In brief, 152 healthy Australian men and women aged 65 years and above were recruited and randomly allocated to either a MedDiet or a habitual diet (HabDiet, control) for 6 months. A total of 137 participants completed the study (MedDiet *n* = 74, HabDiet *n* = 63). Outcome measures included blood pressure, anthropometry (BMI, body weight, abdominal adiposity as measured by dual energy X-ray absorptiometry (DEXA)), waist/hip ratio (WHR), endothelial function, F_2_-isoprostanes (F_2_-IsoP), inflammatory biomarkers, lipids, glucose, insulin, dietary compliance, and cognitive performance and were collected at baseline and at 3 and 6 months of the intervention. Participants attended fortnightly sessions with a study dietitian to ensure adherence was maintained. Dietary intake was assessed with a 3-day weighed food record (WFR) and a food frequency questionnaire (FFQ) [19] at baseline and then during each intervention phase at 2 months and 4 months. The MedDiet was based on the traditional Cretan MedDiet [20] and was rich in fruits, vegetables, extra virgin olive oil, legumes, nuts, grains, and cereals, with lower amounts of red meat, processed foods, and discretionary foods. WFR data were analysed using FoodWorks Professional software (Version 7.0.3016; Xyris Software) to generate grams and servings per day of foods and food groups [17]. These data were used to determine adherence to the dietary prescription and the calculation of DII and E-DII scores. The study was conducted according to the guidelines laid down in the Declaration of Helsinki, and all procedures involving human participants were approved by the Human Research Ethics Committee (22 June 2013, #31163), University of South Australia, Adelaide, Australia. Written informed consent was obtained from all participants before commencement. This trial was registered with the Australian New Zealand Clinical Trials Registry (www.anzctr.org.au (accessed on 8 December 2022)) as ACTRN12613000602729.

### 2.1. Calculating the DII and E-DII Scores

The DII was developed to estimate the inflammatory potential of an overall food pattern and has been described elsewhere [11]. In brief, the DII is a literature-derived dietary index developed from 1943 articles published between 1950 and 2010, which identified 45 food parameters and 6 inflammatory biomarkers. Each food parameter was ranked by its inflammatory potential and given a score if it increased (+1), decreased (−1), or had no effect (0) on the 6 inflammatory markers. Dietary intake data from MedLey participants were applied to the scoring process such that a z-score and centred percentile were calculated, for each food parameter consumed, based on the “standard global mean” and standard deviation. The centred percentile score was then multiplied by the respective “overall food parameter-specific inflammatory effect score”, which was derived from the literature review to obtain the “food parameter-specific DII score”. A sum of each “food parameter-specific DII score” was calculated to obtain an “overall DII^®^ score” for each individual participant in MedLey. DII scores can range from −8.87 to +7.98, with a greater score indicating a more pro-inflammatory diet, and more negative scores indicating a more anti-inflammatory diet. Typically, scores range from a minimum of around −5.5 to +5.5. DII was generated as a “raw” value as well as an adjusted value accounting for total energy consumption, the “energy-adjusted DII (E-DII)”, where an energy-standardised version of the world database was used to calculate scores per 1000 calories [21]. E-DII did not include energy as one of the food parameters. The DII and E-DII are scored similarly and scaled identically; thus, the scores are comparable across studies. 

Food-specific parameters used to calculate the DII that were available from the MedLey study WFR include energy (kJ), carbohydrate (CHO, g), protein (g), total fat (g), monounsaturated fat (MUFA, g), saturated fat (SFA, g), polyunsaturated fat (PUFA, g), omega-6 PUFA (mg), omega-3 PUFA (mg), fibre (g), cholesterol (mg), folic acid (µg), total folate (µg), iron (mg), magnesium (mg), zinc (mg), thiamine (mg), riboflavin (mg), niacin-eq. (mg), vitamin A (RE), vitamin C (mg), vitamin D (µg), vitamin E (mg), β-carotene-eq. (µg), flavanols (mg), flavones (mg), flavonols (mg), flavanones (mg), anthocyanidins (mg), isoflavones (mg), alcohol (g), caffeine (mg), garlic (g), ginger (g), onion (g), black tea (g), green tea (g), pepper (g), thyme/oregano (mg), and rosemary (mg). 

### 2.2. Statistical Analysis

Statistical analyses were performed using SPSS version 21.0 (SPSS Inc., Chicago, IL, USA).

Descriptive statistics were presented as mean ± SD with outcome variables reported as mean ± SEM. Statistical significance was set at *p* < 0.05. Data were checked for normality and outliers; no transformations or exclusions were required. Baseline data from the MedDiet and HabDiet were compared with independent-sample t-tests, and the effects of diet over time were assessed with linear mixed effect models, between groups and within groups, with a group x time interaction term to determine overall differences in effects across time and at each time point. All analyses were performed on both “raw” and “E-DII” models.

#### 2.2.1. Statistical Analysis of Changes in DII and E-DII Scores with Dietary Intervention

The effects of dietary interventions on the DII^®^ scores across time and between intervention groups were assessed using an intention-to-treat analysis (including all participants who commenced the study) using linear mixed effects modelling with an unstructured covariance. The dietary intervention group was the between-subject factor, and time was the repeated within-subject measurement. Where there was a significant main effect, post hoc comparisons were performed with Bonferroni’s adjustments for multiple comparisons to determine differences between group means. 

#### 2.2.2. Statistical Analysis of Intervention Impacts Relating to DII and E-DII Scores

Results from the MedLey study showed significant improvements to cardiovascular risk factors. To assess if there was an association of the change in DII^®^ with these outcomes, the changes from baseline were calculated and Pearson’s correlations applied. Assessments of these correlations were also made after dividing the data into varying obesity categories. Tertiles of BMI were established. Normal weight and overweight categories were considered, where normal weight was categorised as a BMI ≤ 24.9 kg/m^2^, and overweight was ≥25 kg/m^2^, as well as considering the elderly population and categorising normal weight for elderly as BMI < 30 kg/m^2^ and overweight elderly as >30 kg/m^2^. Additionally, data were divided into “normal to elevated blood pressure (SBP < 140 mmHg)” and “elevated blood pressure (SBP ≥ 140 mmHg)” categories for analyses.

#### 2.2.3. Statistical Analysis of Cross-Sectional Relationships of DII^®^ and E-DII with Health Parameters 

Baseline data were examined irrespective of dietary intervention group to determine relationships between DII and body composition (BMI, weight, WHR, and abdominal adiposity), cardiometabolic health (hsCRP, blood cholesterol levels, homeostatic model of insulin resistance (HOMA)), and vascular health (blood pressure, flow mediated dilatation). DII was divided into tertiles to establish low (most anti-inflammatory), medium, and high inflammatory dietary patterns. Univariate ANOVA was used to test for differences in health variables with the DII tertile as the fixed-factor variable. Where there was a significant effect, post hoc comparisons were performed with Bonferroni’s adjustments for multiple comparisons to determine differences between group means.

## 3. Results

Baseline characteristics of the MedLey population are published elsewhere [17,22]. Briefly, participants were, on average, 71 ± 5 years, with a BMI of 26.9 ± 3.9 kg/m^2^. They had an acceptable glucose and lipid profile according to the National Heart Foundation of Australia and Cardiac Society of Australia and New Zealand position statement on lipid management [23] (total cholesterol 5.2 ± 0.9 mmol/L, triglycerides (TGs) 1.2 ± 0.5 mmol/L, HDL cholesterol 1.6 ± 0.4 mmol/L, LDL cholesterol 3.0 ± 0.8 mmol/L, total:HDL cholesterol ratio 3.4 ± 0.9, glucose 5.2 ± 0.6 mmol/L) and high-normal blood pressure (SBP 134 ± 17 mmHg, DBP 83 ± 10 mmHg, heart rate (HR) 64 ± 9 bpm) according to the National Heart Foundation of Australia guideline for the diagnosis and management of hypertension in adults [24].

### 3.1. Dietary Adherence to a MedDiet

Adherence to a MedDiet was calculated using a 15-point adherence score based on the methodology of Trichopoulou [25]. There was no significant difference between adherence scores at baseline between groups (MedDiet 7.1 ± 1.9, HabDiet 7.4 ± 2.4, *p* = 0.41). Following intervention, the MedDiet group had significantly (*p* < 0.001) increased their score to 10.6 ± 1.7 to achieve high adherence, while the HabDiet group remained unchanged with a score of 7.9 ± 2.5 (*p* = 0.235) [26].

### 3.2. Dietary Inflammatory Index

**Figure 1:**
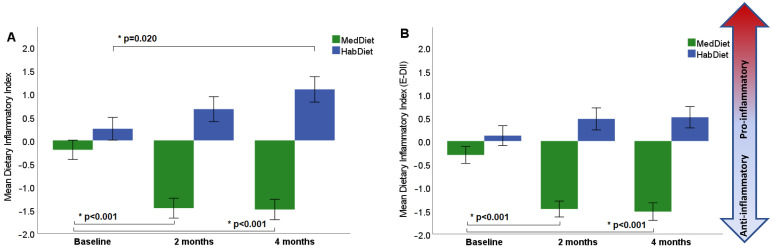
Changes in the dietary inflammatory index during the MedLey intervention: (**A**) raw model of dietary inflammatory index; (**B**) E-DII (dietary inflammatory index adjusted for energy). DII was calculated at 2 and 4 months of the intervention period. A more negative DII score indicates a more anti-inflammatory diet, while a more positive score indicates a more pro-inflammatory diet; * *p* values represent statistical significance determine by LMEM. MedDiet, Mediterranean diet; HabDiet, habitual diet.

There was no significant difference in the DII score at baseline between the MedDiet and HabDiet groups (*p* = 0.159). Following 2 and 4 months of dietary intervention, the MedDiet group had significantly changed to a more anti-inflammatory diet with a reduction in DII score from −0.20 ± 1.84 at baseline to −1.49 ± 1.83 at 4 months (Table 1 and Figure 1a). The HabDiet was unchanged after 2 months (*p* = 0.360) and became more pro-inflammatory after 4 months (*p* = 0.020) (Table 1 and Figure 1a); however, this significance was attenuated when the model was assessed using the energy E-DII model to account for total energy intake (Table 1 and Figure 1b). Compared with HabDiet, the MedDiet resulted in a significant reduction in DII score over 4 months (*p* < 0.001; Table 1).

### 3.3. Cardiometabolic Health Outcomes of the MedLey Trial

Data from the MedLey study has been published elsewhere in detail [27,28] in 2017. In brief, compared with the HabDiet, the MedDiet resulted in a significant reduction in F_2_-IsoP, TGs, and SBP at 3 months and 6 months. Similarly, the MedDiet resulted in improved endothelial function at 6 months, as measured by flow mediated dilatation (FMD), compared with the HabDiet group.

### 3.4. Relationship of DII^®^ to Changes in MedLey Outcome Measures

There was no association found between the changes in cardiometabolic outcomes and the changes in DII, and these results did not change when the population was split into normal weight and overweight categories. We then explored if BP status at baseline may influence cardiometabolic outcomes and divided the data into “normal to elevated SBP (<140 mmHg) (*n* = 125)” and “elevated systolic BP (≥140 mmHg) (*n* = 23)” categories. In participants allocated to the HabDiet group who had elevated SBP (*n* = 11), a significant inverse relationship was seen between the changes in DII^®^ and the changes in F_2_-IsoP (raw model: *p* = 0.021, Pearson correlation −0.682; E-DII model: *p* = 0.09, Pearson correlation −0.740) No other significant relationships between DII or E-DII and cardiometabolic biomarkers were observed. 

### 3.5. Cross-Sectional Relationships of DII^®^ with Health Parameters

**Figure 2:**
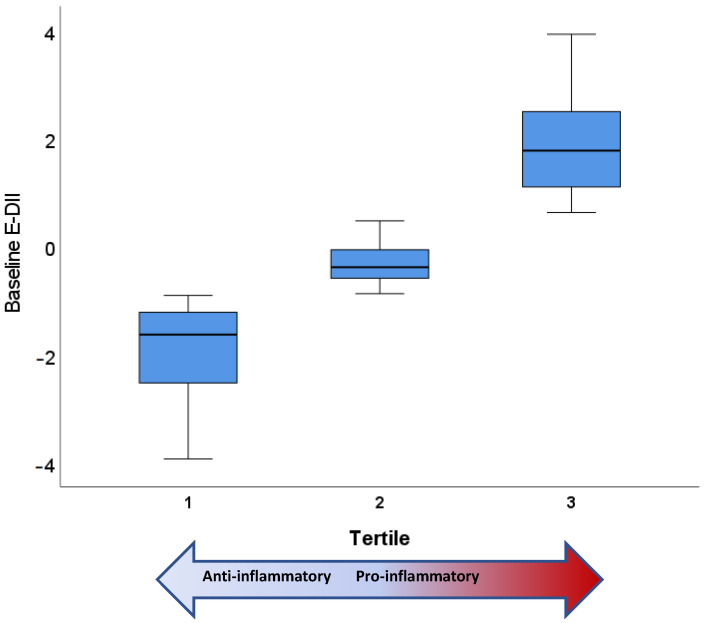
Baseline dietary inflammatory index (adjusted for energy intake) stratified into tertiles. Tertile 1 represents the lowest E-DII, being the most anti-inflammatory, while tertile 3 represents the highest E-DII, being the most pro-inflammatory.

As there was no relationship between the change in cardiometabolic health outcomes of the dietary intervention and DII, baseline data from both groups were combined to explore relationships between DII status and cardiometabolic parameters. DII tertiles were established; the most anti-inflammatory dietary pattern (tertile 1: Low) included DII^®^ scores (raw model) ranging from −5.03 to −0.93 with a mean score of −2.04. The middle tertile (tertile 2: Medium) included DII scores from −0.90 to 0.70 with a mean score of −0.13. The most pro-inflammatory dietary tertile (tertile 3: High) ranged from 0.74 to 4.55, with a mean score of 2.16. For the E-DII^®^ model (Figure 2), the DII tertiles were tertile 1 (Low) from −3.92 to −0.90 with a mean score of −1.90; tertile 2 (Medium) from −0.87 to 0.47 with a mean score of −0.27; and tertile 3 (High) from 0.63 to 3.92 with a mean score of 1.87.

**Figure 3:**
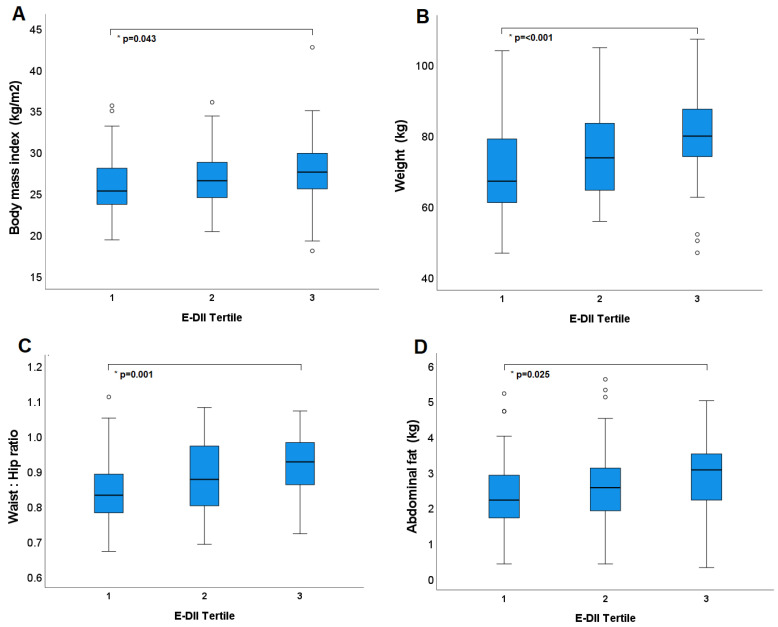
Tertiles of dietary inflammatory index (adjusted for energy) and adiposity at baseline: (**A**) body mass index; (**B**) weight in kilograms; (**C**) waist to hip ratio; (**D**) abdominal fat as determined by DEXA scan. * *p* values represent statistical significance determine by LMEM. Circles outside error bars represent outliers.

There was a significant difference in average baseline BMI between both the lowest and middle DII tertiles and the highest tertile (*p* = 0.001 and *p* = 0.025, respectively), where those with a more pro-inflammatory DII^®^ had higher BMI. When controlling for dietary energy intake (E-DII model), the significant difference between the lowest and highest tertiles was maintained (*p* = 0.04) (Figure 3a).

Similarly, average body weight differed significantly from the two lower tertiles to the highest tertile (*p* = 0.0005, *p* = 0.01, respectively) in the raw model and between the lowest and highest in the E-DII model (*p* < 0.0001) (Figure 3b) (Table 2). WHR was higher in the most pro-inflammatory tertile than the middle tertile (raw model *p* = 0.007) and the most anti-inflammatory tertile (E-DII model *p* = 0.001; Figure 3c, Table 2). There was a significant difference between the lowest and highest tertile groups, when assessing average abdominal fat, in both models (*p* < 0.0001 (raw), *p* = 0.03 (E-DII); Figure 3d).

**Figure 4:**
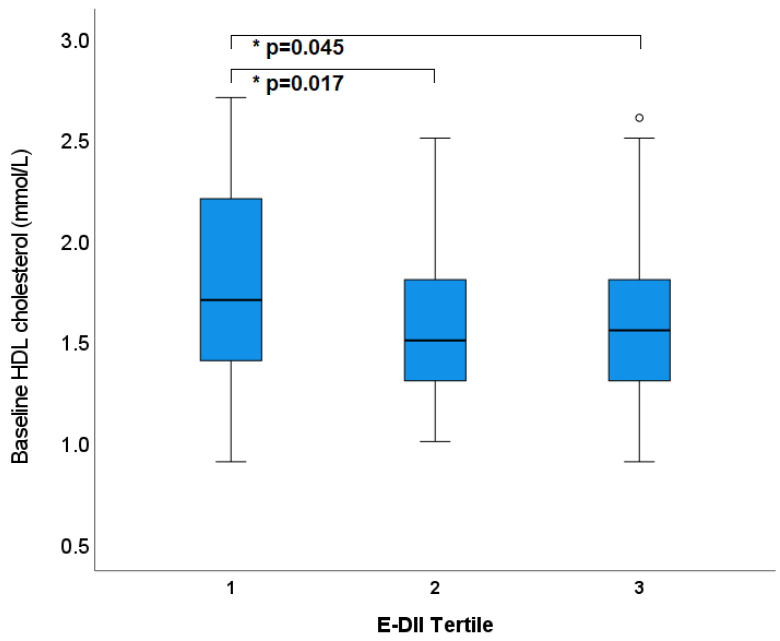
Tertiles of Dietary Inflammatory Index (adjusted for energy) and HDL cholesterol at baseline. * *p* values represent statistical significance determine by LMEM. Circles outside error bars represent outliers.

Blood lipids did not significantly differ across the different tertiles of DII in the raw model. When controlling for energy and total cholesterol, TGs and LDL-C remained non-significant across tertiles; however, HDL-C and consequently total:HDL-C showed significant differences (Figure 4, Table 2). In the most anti-inflammatory tertile, the average HDL-C was 1.78 ± 0.06 mmol/L, which was significantly higher (*p* = 0.02, *p* = 0.04, respectively) than the middle tertile (1.55 ± 0.06 mmol/L) and the most pro-inflammatory tertile (1.57 ± 0.06 mmol/L). 

There was no significant association found between hsCRP, F_2_-IsoP, and FMD and DII^®^ tertiles. 

**Figure 5:**
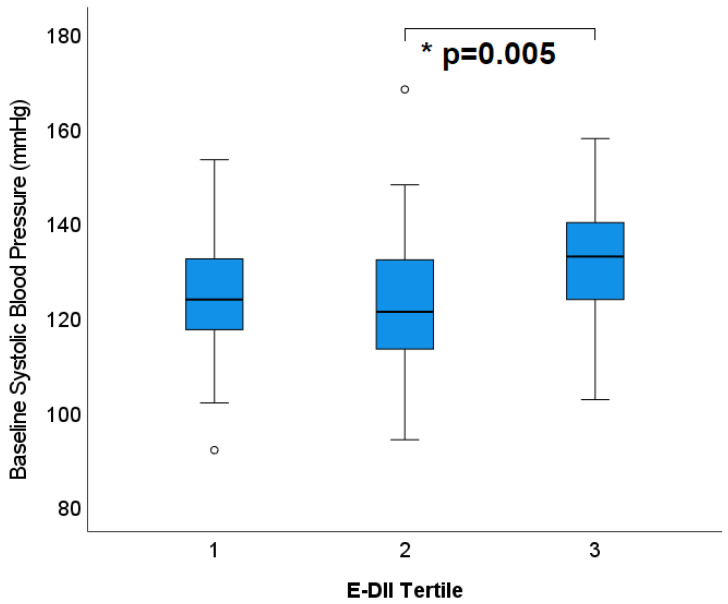
Tertiles of dietary inflammatory index (adjusted for energy) and systolic blood pressure at baseline; * *p* values represent statistical significance determine by LMEM. Circles outside error bars represent outliers.

When analysed with the E-DII model, SBP was significantly higher for those with a more pro-inflammatory DII^®^ (Figure 5) (=0.005). Mean SBP values for the lower tertiles were 125 ± 1.8 and 123 ± 1.9, respectively, while the highest tertile had an average of 131 ± 1.8 mmHg. The raw model, which did not account for dietary energy, did not capture this significant difference (*p* = 0.09). Diastolic BP was unchanged across DII tertiles (*p* = 0.50 (raw), *p* = 0.41 (E-DII)). 

## 4. Discussion

In this randomised dietary intervention trial, we demonstrated that high adherence to a MedDiet over 6 months led to improved DII/E-DII scores and anti-inflammatory activity compared to the HabDiet group in an older Australian cohort. However, no associations between DII/E-DII and cardiometabolic risk factors were found, except for a subset of participants with elevated SBP at baseline. A baseline cross-sectional analysis revealed significant differences between the highest (most inflammatory) and lowest (least inflammatory) E-DII tertiles. Those in the highest tertile of DII had higher average BMI, body weight, WHR, abdominal adiposity, and SBP, and lower HDL-C. No significant associations between hsCRP, F_2_-IsoP, and FMD, and DII/E-DII were found. The mean DII at baseline was −0.10 ± 1.71, which is marginally more inflammatory than that in the Observation of Cardiovascular Risk Factors in Luxembourg (ORISCAV-LUX) study (*n* = 1352; −0.41 ± 1.62) [29] and much less inflammatory than that of American participants in the Seasonal Variation of Blood Cholesterol Study (SEASONS) (0.84 ± 1.99) [30]. These differences may be explained by the overall dietary patterns of these countries, with relatively two thirds of the ORISCAV-LUX participants receiving anti-inflammatory scores, indicating an overall healthier diet than the American participants. Further, although the Australian and American diets follow the more traditional Western dietary patterns, the age of our MedLey participants (>65 years) may explain the lower DII observed, with some studies reporting an inverse relationship between age and DII scores [14,29].

Previous studies have demonstrated the association between MedDiet adherence and DII. A 2018 Australian cohort study including over 40,000 participants reported an inverse relationship between MedDiet adherence and DII scores [31]. Similarly, in participants from the Primary Prevention of Cardiovascular Disease with a Mediterranean Diet trial (PREDIMED) (*n* = 7236), higher MedDiet adherence scores were associated with lower DII scores [32]. To our knowledge, the only intervention study available for reasonable comparison is the AUSMED Heart trial, which investigated the inflammatory potential of a MedDiet vs. a low-fat diet, and its impact on well-known inflammatory biomarkers hsCRP and IL-6 in 56 participants with Coronary Heart Disease in Australia [33]. A significant reduction in DII was observed in the MedDiet group only; however, no significant changes in hsCRP or IL-6 were found [33]. Our findings add to the limited literature investigating the impact of dietary intervention on DII. 

Our analyses reveal an association between dietary inflammatory potential and various parameters of obesity including BMI, abdominal adiposity, waist circumference, and WHR. Cross-sectional and longitudinal studies in the area failed to reach consensus on the topic. In line with our results, in the previously mentioned PREDIMED study, participants in the highest DII quintile had higher average WC and WHR compared to those in the lowest quintile. In female participants, this relationship was also reported with BMI [32]. Ramallal and colleagues reported a significant relationship between DII and a risk of overweight and obesity in their prospective study in the Seguimiento University of Navarra (SUN) cohort (*n* = 7027), with a pro-inflammatory DII being associated with higher annual weight gain [34]. Similarly, in participants from the US National Health and Nutrition Survey (*n* = 17,689) and the SEASONS study (*n* = 519), E-DII and DII scores were positively associated with BMI [35]. Comparable results were reported in cross-sectional populations from Iran (*n* = 6538), Brazil (*n* = 3151), and Australia (*n* = 27,834) [36,37,38]. However, conflicting evidence has also been reported in the literature. In the previously mentioned ORISCAV-LUX study, participants in the highest DII tertile had lower BMI and WC [29]. This was also seen in a cohort of Italian participants (*n* = 20,823) [39]; however, both studies reported that the highest DII groups also had a significantly lower mean age. Further, no associations were found in two similar studies in Iranian women (*n* = 266, *n* = 300), and adults in Indonesia (*n* = 503) and Lebanon (*n* = 331) [40,41,42,43]. These varying results are possibly due to differences in study design, sample size, participant ethnicity, and varying dietary and anthropometric data collection methods. 

At present, the literature supports a significant association between a pro-inflammatory diet, with a higher DII/E-DII, and CVD risk factors, events, and mortality [14,44]. Our study adds to current evidence, with participants in the most anti-inflammatory tertile having higher HDL-C and lower SBP, when controlled for total energy intake. Similar results were reported by Phillips and colleagues in The Cork and Kerry Diabetes and Heart Disease study (*n* = 1992). Additionally, the ORISCAV-LUX study reports that while participants in their highest DII quintile also had lower levels of HDL-C, the mean SBP was lower. Further, Tyrovolos and colleagues reported participants who consumed pro-inflammatory diets to more likely have at least CVD risk factors, including obesity, diabetes, hypertension, and hypercholesterolemia in an analysis of American adults [45]. 

During our initial investigations looking at between-group differences, we identified a small sub-group of participants allocated to the HabDiet group with initial elevated BP at baseline (*n* = 11). Unexpectedly, an inverse relationship was found between DII and F_2_-IsoP in both the DII and E-DII models. These incidental results are not in accordance with the literature or our expectations and should be interpreted with caution. 

Our study has several strengths. First, to our knowledge, this is the first study investigating the effect of a DII lowering dietary intervention on cardiometabolic outcomes in healthy older adults. Second, the use of 3-day weighed food records allows for a more accurate representation of participant diet. The more detailed records allow for the use of 40 of the 45 food parameters used to calculate DII, whereas some previous studies used 20–35 parameters [29,32,34,39]. Therefore, our study more accurately captures the full inflammatory potential of the diet than many previous studies. Third, the use of DEXA to assess abdominal adiposity provides a more precise measure than BMI or WHR, allowing for comprehensive analyses.

Despite its strengths, this study also has limitations. This was not the primary aim of the MedLey study, and with the sample size (*n* = 137) and study length (6 months), the study likely lacked statistical power to identify correlations between DII and the cardiometabolic outcomes observed. Additionally, the study only measured one inflammatory biomarker (hsCRP). While this is a highly sensitive measure of inflammation, it lacks the specificity that additional biomarkers would contribute.

## 5. Conclusions

Our results provide further evidence regarding the relationship between the dietary inflammatory index of a MedDiet and cardiometabolic outcomes. We demonstrated that high adherence to a MedDiet intervention over 6 months significantly lowered mean DII compared to a HabDiet in older Australians. The reduction of the inflammatory load of the diet in this trial suggests a mechanism by which a MedDiet pattern could improve cognitive and cardiovascular health. This suggests that increasing compliance with this diet could be reductive or preventative in the elderly for these and other chronic health conditions, which may be useful for clinical practice. The baseline analyses provide additional affirmation of the relationship between DII and some CVD risk factors. However, results across studies are inconsistent, making consensus challenging. Future studies in larger well-powered samples, with longer intervention periods and more diverse inflammatory biomarkers, are needed to ascertain whether a decrease in DII mediated by a MedDiet intervention is effective in reducing inflammation and CVD risk factors.

## Figures and Tables

**Table 1:** Comparison of dietary inflammatory index score between Mediterranean diet and habitual diet over time.

Mean ± SEM	MedDiet									Diet x Visit Interaction	Diet	Visit
	0 month	2 month	4 month	Change 0–2 month			Change 0–4 month					
	(*n* = 79)	(*n* = 69)	(*n* = 70)	mean change	*p* value	95%CI	mean change	*p* value	95%CI	*p* value	*p* value	*p* value
DII	−0.20 ± 0.22	−1.27 ± 0.22	−1.32 ± 0.22	1.07 ± 0.23	<0.001	0.51, 1.63	1.11 ± 0.26	<0.001	0.49, 1.74	<0.001	<0.001	0.108
E-DII	−0.30 ± 0.19	−1.40 ± 0.20	−1.47 ± 0.20	1.10 ± 0.22	<0.001	0.57–1.63	1.17 ± 0.22	<0.001	0.63, 1.71	<0.001	<0.001	0.034
	**HabDiet**											
	0 month	2 month	4 month	Change 0–2 month			Change 0–4 month					
	(*n* = 71)	(*n* = 67)	(*n* = 65)	mean change	*p* value	95%CI	mean change	*p* value	95%CI			
DII	0.24 ± 0.23	0.62 ± 0.23	0.98 ± 0.23	−0.37 ± 0.24	0.36	−0.21, 0.95	−0.73 ± 0.27	0.02	−1.38, −0.09			
E-DII	0.12 ± 0.20	0.47 ± 0.21	0.54 ± 0.21	−0.35 ± 0.22	0.35	−0.90, 0.19	−0.43 ± 0.23	0.21	−0.99, 0.14			

Showing statistical analysis by linear mixed effects model with a diet x time interaction comparing MedDiet and HabDiet across 3 time points. Mean values ± SEM. DII, dietary inflammatory index; E-DII, dietary inflammatory index adjusted for energy; MedDiet, Mediterranean diet; HabDiet, habitual diet; 95%CI, 95% confidence interval.

**Table 2:** Baseline values for MedLey outcome variables per DII tertile.

	Model	Tertile 1	Tertile 2	Tertile 3	Trend
		Low	Medium	High	(ANOVA)
BMI	1	25.71 ± 0.54 (24.65, 26.76) ^¥^	26.41 ± 0.54 (25.35, 27.47) ^¥^	28.41 ± 0.52 (27.37, 29.45) ^ӿ†^	*p* = 0.001
	2	26.07 ± 0.54 (24.99, 27.14) ^¥^	26.57 ± 0.54 (25.50, 27.64)	27.98 ± 0.55 (26.89, 29.06) ^ӿ^	*p* = 0.040
Weight (kg)	1	70.41 ± 1.77 (66.91, 73.90) ^¥^	72.99 ± 1.77 (69.50, 76.49) ^¥^	80.09 ± 1.73 (76.66, 83.51) ^ӿ†^	*p* < 0.0005
	2	69.76 ± 1.75 (66.30, 73.23) ^¥^	74.39 ± 1.75 (70.92, 77.85)	79.67 ± 1.77 (76.17, 83.17) ^ӿ^	*p* = 0.001
WHR	1	0.88 ± 0.01 (0.85, 0.90)	0.86 ± 0.01 (0.83, 0.88) ^¥^	0.91 ± 0.01 (0.89, 0.94) ^†^	*p* = 0.008
	2	0.85 ± 0.01 (0.82, 0.87) ^¥^	0.88 ± 0.01 (0.85, 0.90)	0.92 ± 0.01 (0.89, 0.94) ^ӿ^	*p* = 0.001
Abdominal fat (kg)	1	2.18 ± 0.15 (1.89, 2.47) ^¥^	2.65 ± 0.15 (2.35, 2.94)	3.05 ± 0.15 (2.77, 3.34) ^ӿ^	*p* = 0.000
	2	2.33 ± 0.15 (2.03, 2.63) ^¥^	2.64 ± 0.15 (2.34, 2.94)	2.91 ± 0.15 (2.61, 3.21) ^ӿ^	*p* = 0.030
HDL-C (mmol/L)	1	1.69 ± 0.06 (1.58, 1.81)	1.68 ± 0.06 (1.56, 1.79)	1.52 ± 0.06 (1.40, 1.64)	*p* = 0.087
	2	1.78 ± 0.06 (1.67, 1.90) ^†¥^	1.55 ± 0.06 (1.43, 1.67) ^ӿ^	1.57 ± 0.06 (1.45, 1.69) ^ӿ^	*p* = 0.010
Total:HDL-C	1	3.25 ± 0.12 (3.00, 3.49)	3.33 ± 0.12 (3.09, 3.75)	3.53 ± 0.12 (3.29, 3.77)	*p* = 0.249
	2	3.07 ± 0.12 (2.83, 3.31) ^†¥^	3.54 ± 0.12 (3.30, 3.78) ^ӿ^	3.49 ± 0.12 (3.25, 3.73) ^ӿ^	*p* = 0.012
SBP (mmHg)	1	124.7 ± 1.9 (121.0, 128.5)	124.3 ± 1.9 (120.5, 128.1)	129.6 ± 1.9 (125.9, 133.3)	*p* = 0.093
	2	124.9 ± 1.8 (121.2, 128.5) ^¥^	122.7 ± 1.9 (119.0, 126.4) ^¥^	131.1 ± 1.8 (127.5, 134.8) ^ӿ†^	*p* = 0.005
DBP (mmHg)	1	70.9 ± 1.2 (68.4, 73.3)	71.8 ± 1.3 (69.4, 74.3)	72.9 ± 1.2 (70.5, 75.3)	*p* = 0.502
	2	71.1 ± 1.2 (68.6, 73.5)	71.3 ± 1.2 (68.9, 73.8)	73.2 ± 1.2 (70.8, 75.6)	*p* = 0.414
HR (bpm)	1	65.0 ± 1.1 (62.9, 67.2) ^†^	68.9 ± 1.1 (66.7, 71.1) ^ӿ^	66.7 ± 1.1 (64.6, 68.8)	*p* = 0.045
	2	66.2 ± 1.1 (64.0, 68.4)	68.3 ± 1.1 (66.1, 70.5)	66.1 ± 1.1 (63.9, 68.3)	*p* = 0.290

Baseline values for MedLey outcome variables presented as mean and SEM with 95% confidence intervals when grouped into tertiles of DII^®^ scores. Tertile 1 contains the most anti-inflammatory DII^®^ scores, while tertile 3 contains the most pro-inflammatory DII^®^ scores. Model 1, raw DII^®^ scores; Model 2, E-DII^®^ scores adjusted for dietary energy intake. *n* = 137. ӿ significantly different to tertile 1, Bonferroni post hoc analysis *p* < 0.05. † significantly different to tertile 2, Bonferroni post hoc analysis *p* < 0.05. ¥ significantly different to tertile 3, Bonferroni post hoc analysis *p* < 0.05.

## Data Availability

Data from the MedLey study contains sensitive participant information. For data access, please contact the University of South Australia Human Research Ethics Committee humanethics@unisa.edu.au, researchintegrity@unisa.edu.au.

## Abbreviations

BMI, body mass index; BP, blood pressure; CHO, carbohydrate; hsCRP, high sensitivity C-reactive protein; CVD, cardiovascular disease; DBP, diastolic blood pressure; DII, dietary inflammatory index; F_2_IsoP, F_2_-isoprostanes; FFQ, food frequency questionnaire; FMD, flow mediated dilatation; HabDiet, habitual diet; HDL-C, high density lipoprotein cholesterol; HOMA, homeostatic model of assessment; HR, heart rate; LDL-C, low density lipoprotein cholesterol; MedDiet, Mediterranean diet; MedLey, Mediterranean diet for cognition and cardiovascular health in the elderly study; MUFA, monounsaturated fatty acid; PUFA, polyunsaturated fatty acid; SBP, systolic blood pressure; SD, standard deviation; SEM, standard error of the mean; SFA, saturated fatty acid; TAG, triglycerides; WFR, weighed food records; WHR, waist to hip ratio.
